# Immune Rejection Mediated by *prf1* and *gzmb* Affects the Colonization of Fat Greenling (*Hexagrammos otakii*) Spermatogonia in Heterotransplantation

**DOI:** 10.3390/ijms25105157

**Published:** 2024-05-09

**Authors:** Xi Zhao, Ying Chen, Rui Li, Yu Men, Kai Yan, Zibin Li, Wenxiu Cai, Yan He, Jie Qi

**Affiliations:** 1 MOE Key Laboratory of Marine Genetics and Breeding, College of Marine Life Sciences, Ocean University of China, 5 Yushan Road, Qingdao 266003, China; enactus_zhaoxic@163.com (X.Z.); 15092067730@163.com (Y.C.); 17854252538@163.com (R.L.); menyu626@163.com (Y.M.); yankai_kai@163.com (K.Y.); lizibin2022@163.com (Z.L.); 21220613019@stu.ouc.edu.cn (W.C.); yanhe@ouc.edu.cn (Y.H.); 2 Key Laboratory of Tropical Aquatic Germplasm of Hainan Province, Sanya Oceanographic Institute, Ocean University of China, Sanya 572000, China

**Keywords:** heterotransplantation, immune rejection, *prf1*, *gzmb*

## Abstract

Fish germ cell transplantation holds great potential for conserving endangered species, improving cultured fish breeds, and exploring reproductive techniques. However, low transplantation efficiency is a common issue in heterotransplantation. This study transplanted fat greenling (*Hexagrammos otakii*) spermatogonia into the testes of spotted sea bass (*Lateolabrax maculatus*) to investigate factors that might affect the colonization and fixation of heterologous transplanted germ cells. Results indicated that transplanted fat greenling spermatogonia cells were successfully detected in the early transplantation phase in spotted sea bass. Their numbers gradually decreased over time, and after 10 days post-transplantation, more than 90% of the transplanted cells underwent apoptosis. Transcriptome sequencing analysis of the testes of spotted sea bass and fat greenling spermatogonia on days 1 and 10 post-transplantation revealed that this apoptosis process involved many immune-related genes and their associated signaling pathways. Acute immune rejection marker genes *prf1* and *gzmb* were detected in the spotted sea bass testes, while immune tolerance genes *lck* and *zap-70* were expressed in the fat greenling spermatogonia. Additionally, differential expression of *prf1* and *gzmb* genes was screened from spotted sea bass, with experimental evidence indicating that PRF1 and GZMB protein from spotted sea bass primarily induce apoptosis in transplanted fat greenling spermatogonia via the mitochondrial apoptosis pathway, at the protein level. This suggests that the difficulties in heterotransplantation are primarily related to acute immune rejection, with PRF1 and GZMB playing significant roles.

## 1. Introduction

Fish germ cell transplantation (GCT) has emerged as a novel biotechnological approach. Donor-derived germ cells can be transplanted into the gonads of different individuals or even different species, enabling the recipients to produce gametes that originate from the donors [1]. Adopting GCT technology, researchers successfully transplanted primordial germ cells (PGCs) from rainbow trout (*Oncorhynchus mykiss*) into the abdominal cavities of newly hatched masu salmon (*Oncorhynchus masou*) larvae, leading to the surrogate production of rainbow trout offspring [2,3]. This marked the first successful case of surrogate reproduction in fish. To date, GTC has been successfully implemented in various fish species, including zebrafish (*Danio rerio*) [4], Nile tilapia (*Oreochromis mossambicus*) [5], pejerrey (*Odontesthes bonariensis*) [6], sterlet (*Acipenser ruthenus*) [7], and common carp (*Cyprinus carpio*) [8]. Due to the limited number of PGCs and the complexity of their extraction, spermatogonia, which are more abundant and easily obtained, have been increasingly utilized for transplantation in recent studies. The limited number of PGCs in fish and the complexity of their extraction process contrast with the relatively higher abundance and ease of obtaining spermatogonia. Transplanted spermatogonia retain significant stem cell activity, capable of producing sperm in a testicular environment and oocytes in an ovarian setting [9,10]. Consequently, recent studies have increasingly utilized spermatogonia for transplantation purposes [11,12].

Transplanted cells often encounter difficulties in engraftment, primarily due to immune responses [13]. Cell surface proteins encoded by the genes of the major histocompatibility complex (MHC) or human leukocyte antigens (HLA) determine the fate of transplanted organs or cells. These proteins include at least six functional class I molecules (HLA-A, B, C, E, F, and G) and three groups of class II antigens (HLA-DR, DQ, and DP) [14]. Class I antigens, which are more broadly distributed, are present on virtually all nucleated cells [15,16]. As cell surface glycoproteins, they serve as recognition units, determining whether transplants are subject to attack by cytotoxic T lymphocytes [17]. Therefore, immune rejection is a potential limitation in spermatogonia transplantation, affecting the success of male germ cell transplantation [18].

The fat greenling, an economically important cultured fish species, exhibits distinct seasonal variations in testicular development [19]. During the regression stage of the testis, the main reproductive cells comprise spermatogonia and partially un-ejected sperm [20]. Moreover, a spermatogenic quiescent period, lasting up to four months, occurs during the repetitive spermatogenesis in fat greenling testes, during which the seminiferous tubules are filled with spermatogonia (unpublished data). Therefore, the fat greenling represents a suitable experimental model for spermatogonia research. Spotted sea bass, also known as the Chinese sea bass, is a euryhaline species widely distributed in the northwest Pacific [21]. With its superior meat quality, high economic value, and promising aquaculture potential, the spotted sea bass holds an important position in the aquaculture industry. Due to the larger size of sexually mature individuals, which facilitates manipulation, the spotted sea bass is considered a suitable recipient to validate the issues and challenges present in heterotransplantation.

This study aims to investigate the engraftment efficiency of cross-species spermatogonia transplantation and the factors that may affect successful engraftment. Further, this research focuses on the occurrence of immune rejection responses in the testis of spotted sea bass post-transplantation, and the function of immune-related genes such as *prf1* and *gzmb* in this process, thereby providing a scientific basis for addressing immune rejection issues in cross-species spermatogonia transplantation.

## 2. Results

### 2.1. Transplantation of Fat Greenling Spermatogonia into Busulfan-Treated Spotted Sea Bass

**Figure 1:**
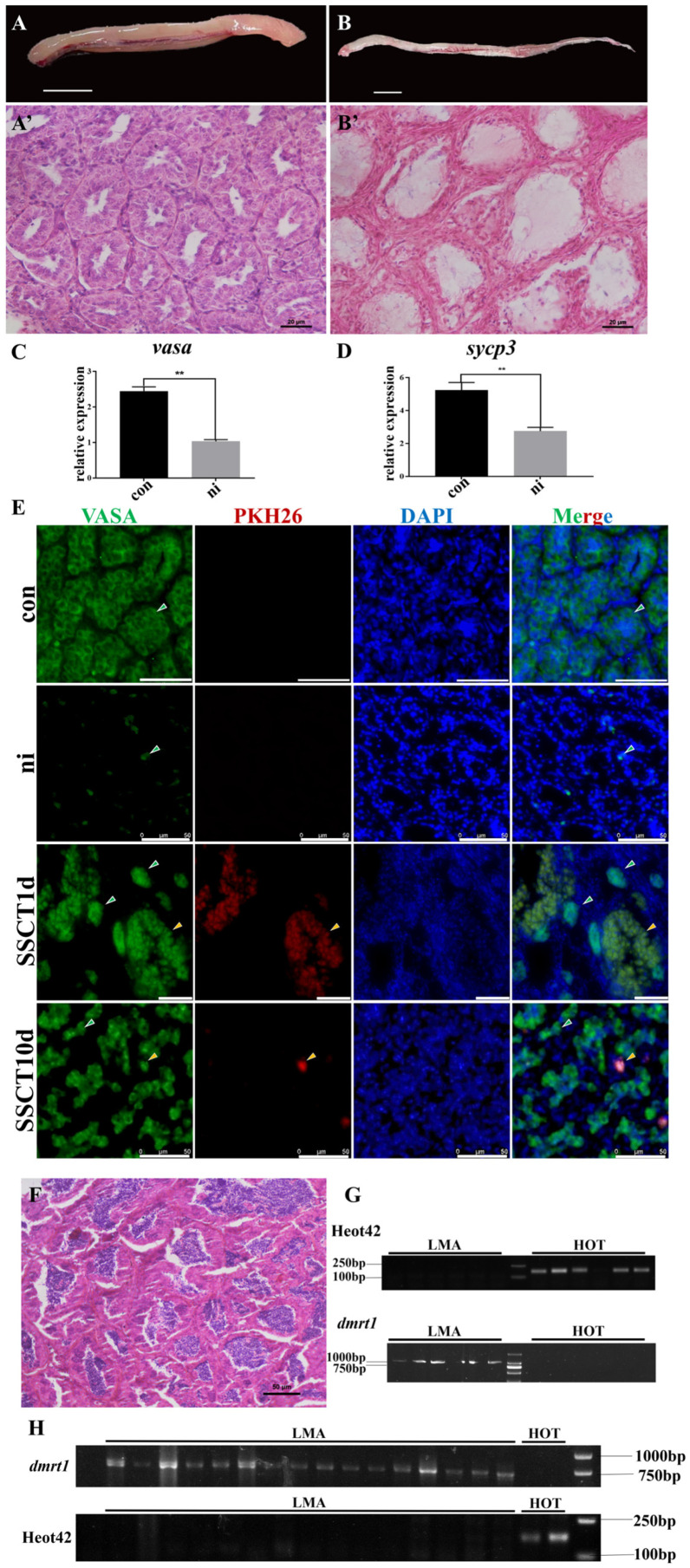
Transplantation of fat greenling spermatogonia into busulfan-treated spotted sea bass testes. (**A**,**A’**) Morphology and histological sections of testes from the control group, (**B**,**B’**) morphology and histological sections of testes from the busulfan-treated group. Scale for testis images, 1 cm; scale for testis sections, 20 μm. (**C**,**D**) Expression changes of *vasa* and *sycp3* genes in testes from the control group (con) and busulfan-treated group (ni). **: *p* < 0.01. (**E**) Co-localization of VASA protein and PKH26 in the testis under different conditions: con, ni, SSCT1d, and SSCT10d. Yellow arrows indicate cells co-localizing VASA and PKH26, while green arrows denote cells expressing VASA only. Scale bar, 50 μm. (**F**) Histological sections of spotted sea bass testis during the breeding season and H and E staining. Scale bar, 50 μm. (**G**) PCR validation of fat greenling microsatellite sequence Heot42 primers and spotted sea bass *dmrt1* gene primers. (**H**) Verification of fat greenling DNA in spotted sea bass testes after transplantation.

To obtain male sterile recipients, we treated the testes of spotted sea bass with busulfan. Morphological and histological observations were conducted on testes from both the control group and the busulfan-treated group. Morphologically, testes from the control group appeared pale yellow and were well formed, whereas testes from the busulfan-treated group were milky white and exhibited a more elongated tissue structure (Figure 1A,B). Histological section results indicated that the testes of the control group were in the spermatogonia proliferation and differentiation stage, with a relatively intact testicular structure, and spermatogonia were the main cells within the seminiferous tubules (Figure 1A’). In contrast, the testes of the busulfan-treated group exhibited prominent cavities, with only a few germ cells observed at the periphery of the seminiferous tubules (Figure 1B’). The qRT-PCR results for the spermatogonia marker gene *vasa* and the spermatocyte marker gene *sycp3* showed a significant reduction in the expression levels of both *vasa* and *sycp3* following busulfan treatment (Figure 1C,D). These findings demonstrate that busulfan treatment significantly damages the testicular tissue of spotted sea bass, leading to a substantial loss of reproductive cells.

To verify the success of spermatogonia transplantation, the con, ni, SSCT1d, and SSCT10d groups were selected for examination through co-localization tracking of the transplanted spermatogonia using VASA protein and PKH26 (Figure 1E). The results demonstrated that in the con group, the seminiferous tubules were full, consisting entirely of VASA^+^ spermatogonia with no PKH26 signal observed. In the ni group, a significant reduction in VASA^+^ cells was observed, along with noticeable vacuoles within the seminiferous tubules and, again, no PKH26 signal was present. In the SSCT1d group, a large aggregation of VASA^+^ cells were detected, with most of these VASA^+^ cells also displaying the PKH26 signal (indicated by yellow arrows), and a minority showing only the VASA^+^ signal (indicated by green arrows). In the SSCT10d group, cells co-localizing VASA and PKH26 were similarly detected, although the overall cell count was reduced. Thus, it is evident that fat greenling spermatogonia were successfully transplanted into the testes of spotted sea bass, with the transplanted cells still observable 10 days after transplantation.

Subsequently, sperm maturation was induced in the transplanted spotted sea bass, followed by sampling and histological observation of the post-induction testes. The sectioning results revealed the presence of apparent sperm within the testes (Figure 1F), yet the overall morphology of the testes was smaller, with irregular seminiferous tubule shapes, suggesting testicular dysgenesis due to repeated busulfan injections. Further, the specificity of the fat greenling microsatellite sequence Heot42 primers and the spotted sea bass *dmrt1* gene primers were verified. The results demonstrated that microsatellites and genetic markers exhibit species specificity (Figure 1G). Thus, it was concluded that the Heot42 primers are suitable for detecting fat greenling DNA within spotted sea bass. When amplifying the Heot42 sequence from DNA samples of 16 transplanted spotted sea bass testes, no target bands were detected (Figure 1H). This outcome indicates that, during the breeding season, the germ cells of fat greenling were not detected in the testes of spotted sea bass. This suggests obstacles in the colonization process of fat greenling spermatogonia within the testes of spotted sea bass.

### 2.2. Apoptosis of Transplanted Spermatogonia

**Figure 2:**
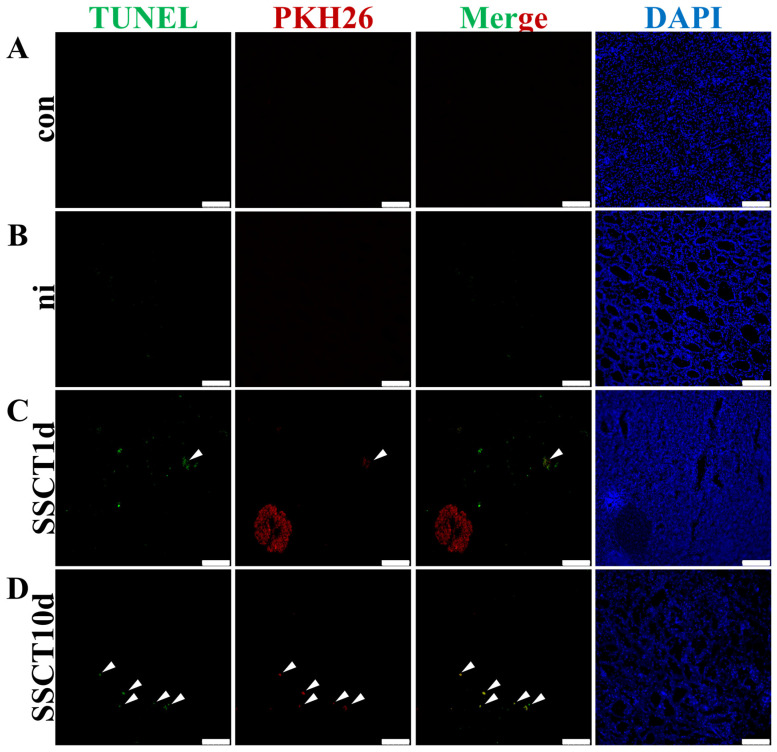
Detection of apoptosis levels in fat greenling spermatogonia after heterotransplantation. Co-localization of TUNEL and PKH26 in the testes of the (**A**) con, (**B**) ni, (**C**) SSCT1d, and (**D**) SSCT10d. White arrows indicate apoptotic spermatogonia of fat greenling. Scale bar, 100 μm.

In the aforementioned experimental results, a significant decrease in the number of transplanted spermatogonia was observed on day 10 after transplantation compared to day 1. To investigate the cause of this decrease in cell number, the apoptosis level of testicular cells before and after transplantation was assessed. The experimental results revealed no significant TUNEL signal in the testes of both the con and ni groups. In the SSCT1d group, weak apoptotic signal was detected in a few of the transplanted cells (Figure 2, indicated by white arrows), while in the SSCT10d group, over 90% of the transplanted cells exhibited a significant apoptotic signal (Figure 2, indicated by white arrows). This indicates that, after 10 days of transplantation, the majority of spermatogonia transplanted into the testes of spotted sea bass had undergone apoptosis, failing to achieve colonization of spermatogonia.

### 2.3. Differentially Expressed Gene Analysis and Functional Enrichment before and after Transplantation

**Figure 3:**
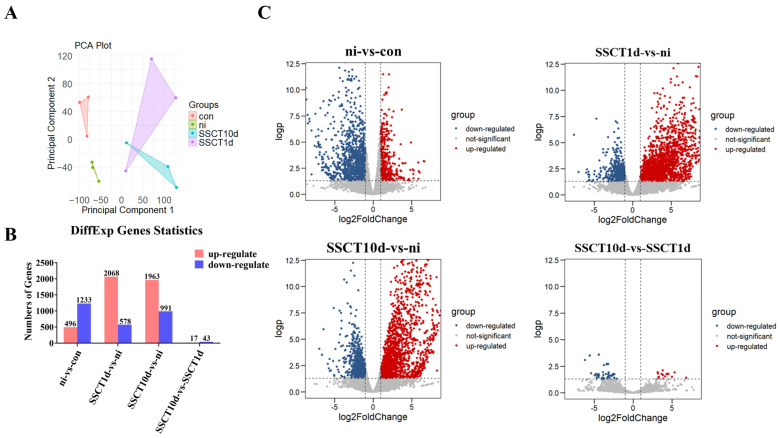
PCA and differential gene expression analysis of spotted sea bass testes before and after heterotransplantation. (**A**) PCA of gene expression profiles in the con, ni, SSCT1d, and SSCT10d. (**B**) Statistical count of differentially expressed genes in comparison groups: ni-vs.-con, SSCT1d-vs.-ni, SSCT10d-vs.-ni, and SSCT10d-vs.-SSCT1d. (**C**) Volcano plots of all genes in comparison groups: ni-vs.-con, SSCT1d-vs.-ni, SSCT10d-vs.-ni, and SSCT10d-vs.-SSCT1d. Blue dots represent downregulated genes, red dots represent upregulated genes, and gray dots represent genes with no significant difference in expression.

To elucidate the reasons behind the challenges in spermatogonia colonization after transplantation, RNA from testes of the con, ni, SSCT1d, and SSCT10d groups was extracted, sequenced, and analyzed. Consequently, 16,638 reads were obtained in the con group, 16,434 reads in the ni group, 16,613 reads in the SSCT1d group, and 16,557 reads in the SSCT10d group, with the con group exhibiting the highest number of reads among the four groups. Prior to differential gene analysis, the separation between groups was assessed. PCA results (Figure 3A) revealed clear separation between the con and ni groups, with good parallelism within each group. A slight overlap between the SSCT1d and SSCT10d groups suggested a closer expression pattern between these two groups. Subsequent identification and statistical analysis of DEGs among the four groups showed that, compared to the con group, the ni group had 496 upregulated genes, 1233 downregulated genes, and 14,082 genes with no significant change (Figure 3B,C). In comparison to the ni group, the SSCT1d group had 2068 upregulated genes, 578 downregulated genes, and 13,165 genes with no significant change; the SSCT10d group had 1963 upregulated genes, 991 downregulated genes, and 12,857 genes with no significant change. Compared to the SSCT1d group, the SSCT10d group had 17 upregulated genes, 43 downregulated genes, and 16,764 genes with no significant change.

**Figure 4:**
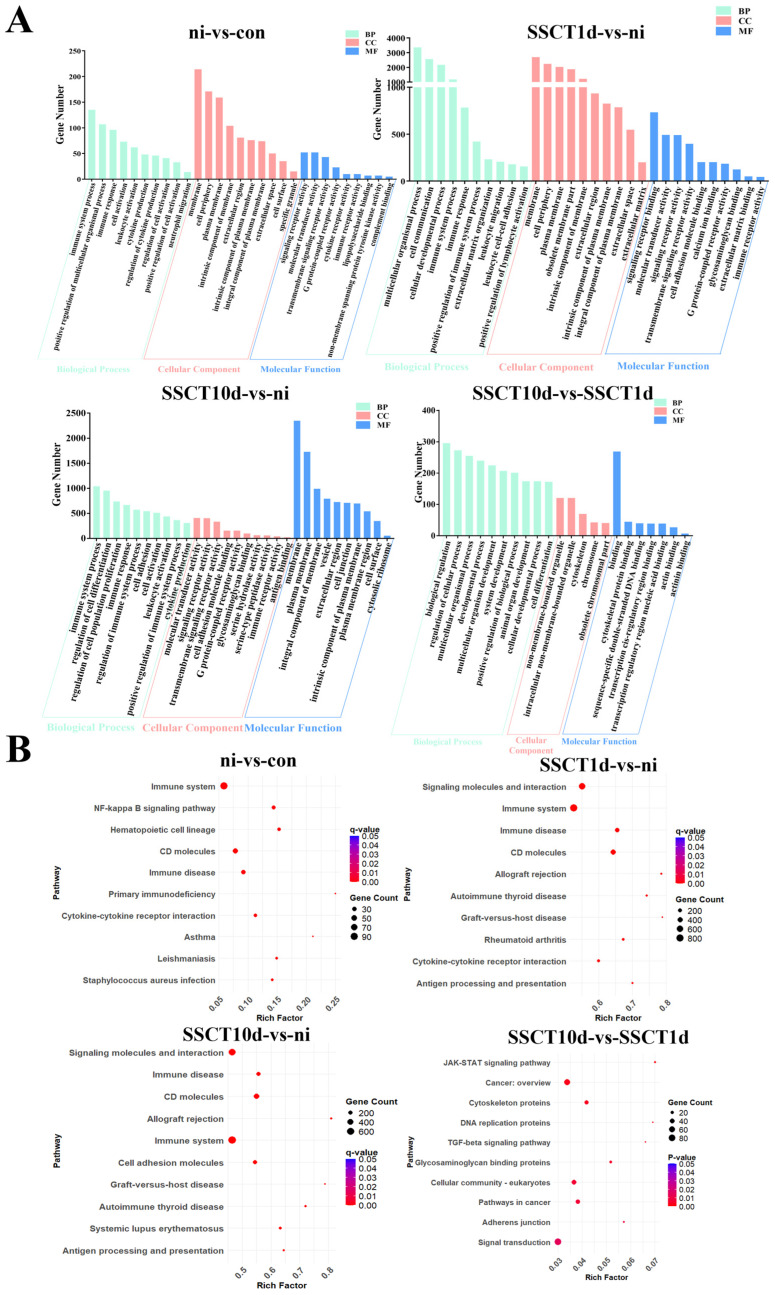
GO and KEGG enrichment analysis of differentially expressed genes in spotted sea bass testes before and after heterotransplantation. GO (**A**) and KEGG (**B**) enrichment analysis of differentially expressed genes in comparison groups ni-vs.-con, SSCT1d-vs.-ni, SSCT10d-vs.-ni, and SSCT10d-vs.-SSCT1d.

DEGs in the comparison groups of ni-vs.-con, SSCT1d-vs.-ni, SSCT10d-vs.-ni, and SSCT10d-vs.-SSCT1d were subjected to GO enrichment and KEGG pathway analyses. The results of the GO enrichment (Figure 4A) demonstrated a significant accumulation of immune-related terms in the comparison groups of ni-vs.-con, SSCT1d-vs.-ni, and SSCT10d-vs.-ni, indicating that busulfan treatment and spermatogonia transplantation can both cause immune responses. The KEGG enrichment results (Figure 4B) revealed an extensive enrichment of disease- and immune-related pathways in the ni-vs.-con comparison group, such as the immune system, NF–κB signaling pathway, and immune diseases, further suggesting that busulfan stimulates an immune response. The pathways enriched in the SSCT1d-vs.-ni and SSCT10d-vs.-ni comparison groups were similar, also showing a significant presence of immune-related pathways, such as the immune system and immune diseases. Additionally, pathways related to immune rejection were enriched, including antigen processing and presentation, allograft rejection, graft-versus-host disease, and autoimmune disease pathways. In the SSCT10d-vs.-SSCT1d comparison group, pathways related to the cytoskeleton, DNA replication proteins, and cancer-related pathways were enriched. The KEGG enrichment results indicate a significant immune rejection response in the early stages after transplantation, which could significantly impact the colonization of spermatogonia. Furthermore, to validate the accuracy of the transcriptome data, genes which differentially expressed between the con group and the ni group were selected for quantitative validation. The results demonstrated that the expression trends of most genes during different developmental stages were consistent with the transcriptome data (Figure S1), thereby indicating that the transcriptome sequencing and subsequent analytical methods were relatively accurate.

### 2.4. Upregulation of Immune-Rejection-Related Genes and Immune Cell Marker Genes after Transplantation

**Figure 5:**
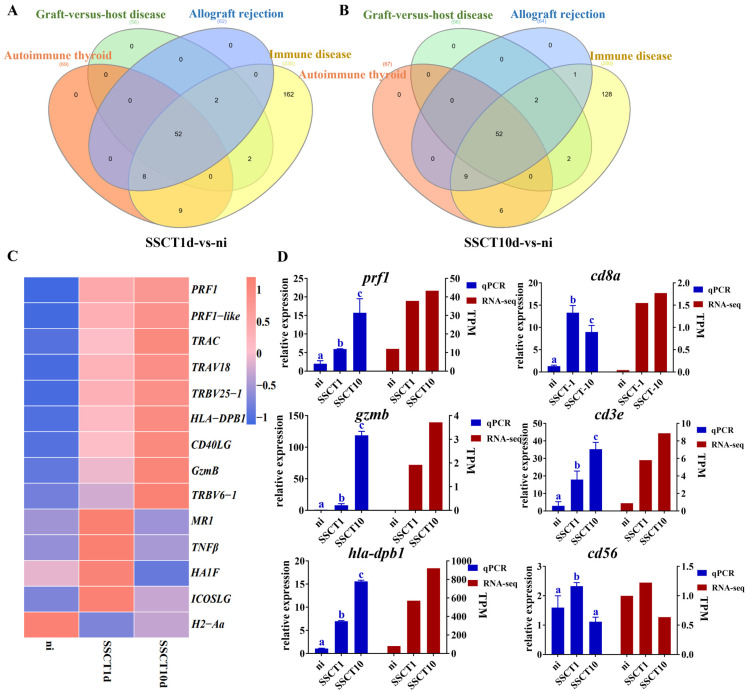
Screening and validation of immune-rejection-related gene expression in spotted sea bass testes after heterotransplantation. (**A**,**B**) Venn diagrams of differentially expressed genes related to autoimmune thyroid disease, allograft rejection, graft-versus-host disease, and immune diseases in comparison groups SSCT1d-vs.-ni and SSCT10d-vs.-ni. (**C**) Heatmap of differentially expressed genes. (**D**) Expression changes of genes *prf1*, *hla-dpb1*, *gzmb*, *cd8a*, *cd3e*, and *cd56* in ni, SSCT1d, and SSCT10d groups. Different letters indicate significant differences between groups (*p* < 0.05).

In the differential expression analysis, both the SSCT1d-vs.-ni and SSCT10d-vs.-ni comparison groups showed enrichment in signal pathways related to immune rejection reactions, such as autoimmune thyroid disease, allogeneic transplant rejection, graft-versus-host disease, and immune diseases. Therefore, the genes selected from the aforementioned four signaling pathways in the SSCT1d-vs.-ni comparison group were intersected, attempting to identify differentially expressed genes involved in all four signaling pathways. A total of 52 differentially expressed genes were finally identified (Figure 5A). Similarly, in the SSCT10d-vs.-ni comparison group, genes differentially expressed across the four pathways were also selected. An intersection of the genes ultimately selected from both groups yielded 14 distinct genes. The expression levels of these genes were statistically analyzed and visualized in a heatmap, revealing significant upregulation of genes within the related pathways post-transplantation, including *prf1*, *prf1-like*, *trac*, *trav18*, *trbv25-1*, *hla-dpb1*, *cd40lg*, *gzmb*, and *trbv6-1* (Figure 5B). Subsequent quantitative validation of *prf1*, *hla-dpb1*, and *gzmb* genes showed a significant increase in expression levels after transplantation (Figure 5C). Considering the close relationship between the immune rejection process and the activity of immune cells, the expression changes of cytotoxic T lymphocyte marker gene *cd8a*, T lymphocyte marker gene *cd3e*, natural killer cell marker gene *cd56*, and T-helper cell marker gene *cd4* were also examined. After day 1 of transplantation, significant increases in the expression levels of *cd56*, *cd3e*, and *cd8a* were observed (Figure 5C), indicating a substantial rise in the number of natural killer cells, T lymphocytes, and cytotoxic T lymphocytes within the testes. Furthermore, after 10 days of transplantation, an additional increase in *cd3e* gene expression suggested a continuous rise in T lymphocyte numbers from day 1 to day 10. However, we did not detect the expression of the *cd4* gene in the testes of spotted sea bass. These experimental results indicate that the expression of genes related to immune rejection and immune cell marker genes significantly increases in the testes of spotted sea bass post-transplantation, suggesting that spermatogonia from the donor may be subject to immune attack by the spotted sea bass recipient.

**Figure 6:**
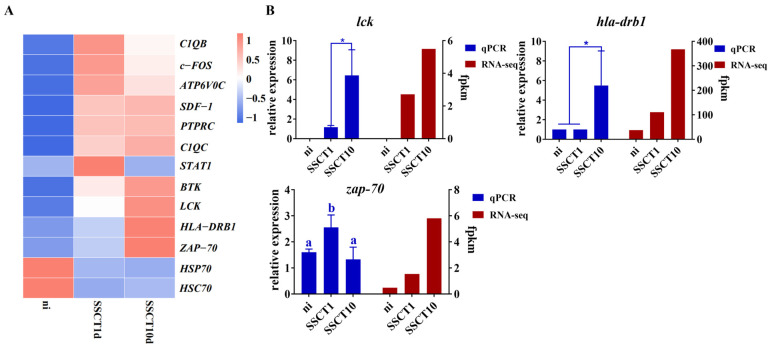
Screening and validation of immune-related differential genes in spermatogonia of fat greenling after heterotransplantation into spotted sea bass testes. (**A**) Heatmap of immune-related differential genes. (**B**) Expression changes of *lck*, *zap-70*, and *hla-drb1* genes in the ni, SSCT1d, and SSCT10d groups. *: *p* < 0.05. Different letters indicate significant differences between groups (*p* < 0.05).

Additionally, we analyzed the expression pattern of fat greenling spermatogonia transplanted into the spotted sea bass. For the analysis of differentially expressed genes in spermatogonia after transplantation, immune-related signaling pathways were also enriched (Figure S2). Statistical analysis of these differential genes identified genes associated with immune responses, and their expression levels were quantified. It was found that the expression of *c1qb*, *c-fos*, *atp6v0c*, *sdf1*, *ptprc*, *c1qc*, *stat1*, *btk*, *lck*, *hla-drb1*, and *zap-70* genes significantly increased post-transplantation (Figure 6A). Immune tolerance genes *lck* and *zap-70*, along with antigen-presenting gene *hla-drb1*, were further selected for quantitative validation. The quantitative results showed significant increases in the expression of *lck*, *zap-70*, and *hla-drb1* genes in spermatogonia after transplantation (Figure 6B). These findings further confirm that transplanted spermatogonia experience immune rejection reactions originating from the donor.

### 2.5. Analysis of Expression Patterns of prf1 and gzmb Genes and Protein Interaction

**Figure 7:**
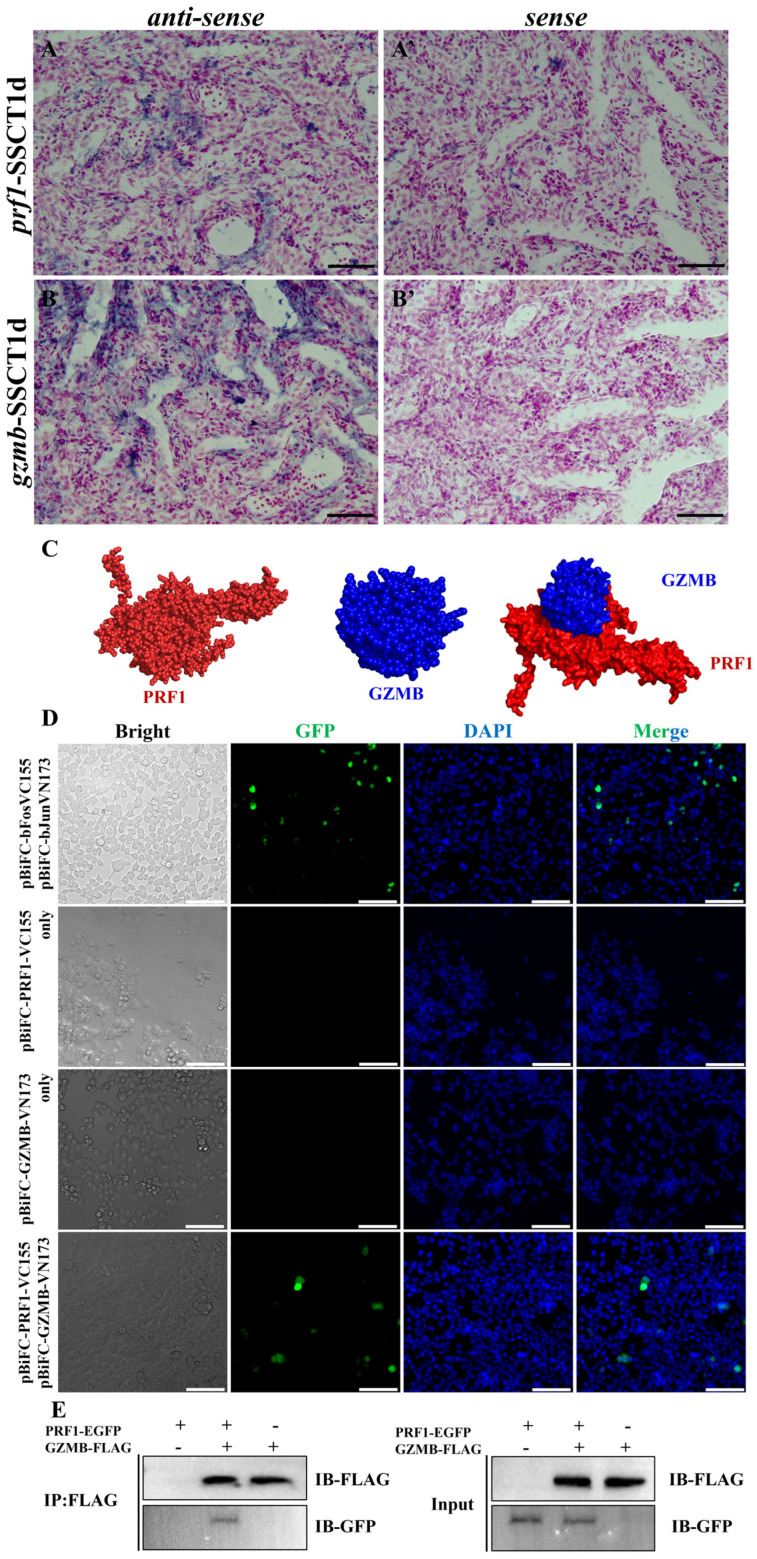
Analysis of expression patterns of *prf1* and *gzmb* genes and protein interaction. (**A**,**A’**) Localization of the *prf1* gene in the testis, one day post-transplantation. (**B**,**B’**) Localization of the *gzmb* gene in the testis, one day post-transplantation; scale bar, 25 μm. (**C**) Structural prediction and molecular docking analysis of PRF1 and GZMB proteins. (**D**) BiFC validation of PRF1 and GZMB interaction; scale bar, 100 μm. (**E**) Co-IP validation of PRF1 and GZMB interaction.

In the aforementioned experimental results, it was found that the expression levels of *prf1* and *gzmb* genes significantly increased in the testes of spotted sea bass after transplantation. To further investigate the role of *prf1* and *gzmb* genes in the immune rejection process of spotted sea bass, the expression patterns of *prf1* and *gzmb* genes were analyzed. Initially, in situ hybridization was conducted to locate the expression of *prf1* and *gzmb* genes in the testes after transplantation (Figure 7A,B). The results of in situ hybridization indicated that *prf1* and *gzmb* were expressed at the periphery of the seminiferous tubules in the testes one day post-transplantation. Subcellular localization results showed that PRF1 was mainly expressed in the cytoplasm, while GZMB was expressed both in the nucleus and cytoplasm (Figure S3). To verify the interaction between the two proteins, the SWISS-MODEL website (https://swissmodel.expasy.org/ (accessed on 12 September 2023)) was used to predict the three-dimensional structure of spotted sea bass PRF1 and GZMB proteins, and the H-DOCK website (http://hdock.phys.hust.edu.cn/ (accessed on 13 September 2023)) was utilized for molecular docking analysis, predicting an interaction between PRF1 and GZMB proteins in spotted sea bass (Figure 7C). BiFC experiment results demonstrated that no gfp fluorescent signal was detected in the groups transfected with either pBiFC-PRF1-VC155 or pBiFC-GZMB-VN173 alone; a significant gfp fluorescent signal was observed in the co-transfection group, though lower than the positive control group. Moreover, in the co-transfection group, the positive signal was primarily expressed in the cytoplasm (Figure 7D), suggesting a significant protein interaction between spotted sea bass PRF1 and GZMB. Finally, this result was further verified by Co-IP experiments. Co-IP results showed that the FLAG antibody could bind to the PRF1-EGFP recombinant protein (Figure 7E), indicating a direct protein interaction between PRF1 and GZMB in spotted sea bass.

### 2.6. Spotted Sea Bass Testes’ PRF1 and GZMB Induce Apoptosis in Fat Greenling Spermatogonia

**Figure 8:**
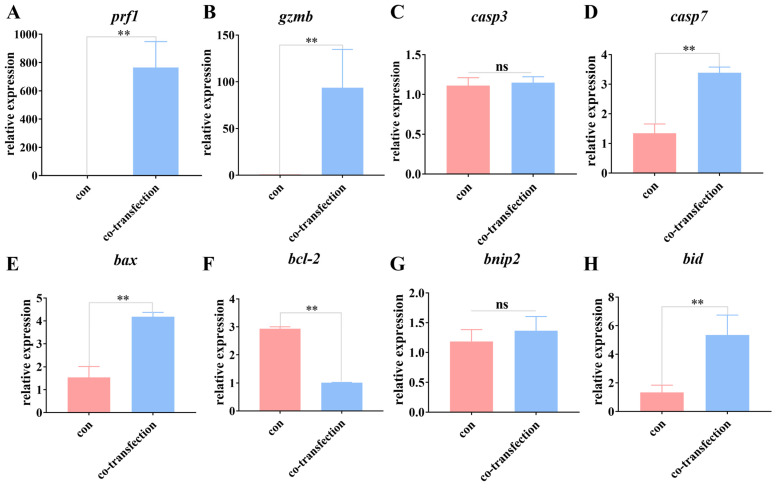
Relative expression of *prf1*, *gzmb*, and apoptosis-related genes in the spermatogonia after transfection. Relative expression of genes *prf1* (**A**), *gzmb* (**B**), *casp3* (**C**), *casp7* (**D**), *bax* (**E**), *bcl-2* (**F**), *bnip2* (**G**), and *bid* (**H**) in spermatogonia of fat greenling in the control and co-transfection groups. ns: *p* > 0.05; **: *p* < 0.01.

To further validate the impact of spotted sea bass PRF1 and GZMB on fat greenling spermatogonia, PRF1-pEGFP-N1 and GZMB-pcDNA3.1-3×FLAG vectors were co-transfected into fat greenling spermatogonia. The efficiency of transfection was first validated by qRT-PCR. Quantitative results for *gzmb* and *prf1* genes indicated no expression of spotted sea bass in non-transfected cells, while their expression was detectable in transfected fat greenling spermatogonia, confirming successful expression of the vectors in fat greenling spermatogonia (Figure 8). Subsequently, apoptosis-related genes *casp3*, *casp7*, *bax*, *bcl-2*, *bnip2*, and *bid*, which were identified to be expressed in spermatogonia after transplantation from transcriptome data, were quantitatively analyzed in the cells after transfection (Figure 8). The quantitative results showed no significant change in the expression of *casp3* and *bnip2* genes, whereas the expression of *bax*, *casp7*, and *bid* significantly increased, and the expression of *bcl-2* gene decreased. Thus, it is demonstrated that spotted sea bass PRF1 and GZMB can induce apoptosis in fat greenling spermatogonia.

## 3. Discussion

Transplantation of germ cells and testicular tissue serves as a promising approach for the propagation of endangered and economically important species [22]. However, the production of functional sperm through testicular transplantation is currently restricted to isogeneic lineages or autologous transplants, while allogeneic and xenogeneic transplants face rejection by the recipient immune system in fish [23,24]. Immune rejection involves a specific cell-mediated process, wherein transplanted tissues and cells are recognized and destroyed by the immune system of the recipient [25]. Consequently, immune rejection is considered a major limiting factor in transplantation research, necessitating the use of conspecific or immunodepleted fish to avoid rejection and achieve long-term engraftment [26]. However, the relationship between immune rejection and the low efficiency of germ cell transplantation remains unclear in current studies, particularly in the context of germ cell transplantation in fish.

In this study, we primarily investigated the potential issues and challenges that may arise during the process of spermatogonia heterotransplantation. In current research on spermatogonia transplantation in fish, the success rate of transplantation is primarily influenced by factors such as the number and purity of transplanted cells, the age of the recipient fish, and the degree of infertility of the recipient fish [27]. However, there is limited research specifically addressing the impact of immune responses on the success rate of spermatogonia transplantation. In our study, during the early stages of transplantation, an unexpected observation was made of extensive apoptosis among the transplanted cells, and significant immune-rejection-related signaling pathways were enriched in the spotted sea bass testicular transcriptome, such as allogeneic transplant rejection and graft-versus-host disease. This indicated that an immune rejection response may occur in the spotted sea bass testes during the early stage of transplantation. The fat greenling belongs to the order Scorpaeniformes, while the spotted sea bass belongs to the order Perciformes. These taxonomic differences indicate that the two species are phylogenetically distinct. This divergence can lead to immune rejection during transplantation, as each species’ immune system is adapted to recognize and respond to foreign cells from even distantly related species, resulting in transplantation rejection.

Immune rejection involves the participation of various immune cells. In studies related to mammals, T cell activation is central to graft rejection [28]. Research indicates that the CD3 molecule, expressed as a co-receptor in mature T lymphocytes, can serve as a surface marker for T lymphocytes [29]. CD8 is a marker gene for cytotoxic T cells, while CD4 is a marker gene for T-helper cells [30]. Shibasaki et al. revealed that T-helper cells are the initial attackers against the transplanted cells, followed by cytotoxic T cells and B cells, collaborating with T cells to accomplish the immune rejection process [31]. The expression levels of T cell marker genes, except for CD4, were upregulated in the testicular transcriptome post-transplantation, indicating that immune cells were active in the spotted sea bass testes following transplantation, and the rejection response was strong.

Typically, perforin (PRF1) and granzyme B (GZMB) serve as markers of acute immune rejection. They are critical factors secreted by cytotoxic T lymphocytes and natural killer cells, acting synergistically to kill cells [32]. PRF1 directly damages the cell membrane of the host cell and facilitates the transfer of GZMB into the cell, after which GZMB induces apoptosis in the target cell [33]. Studies have shown that in patients with acute immune rejection following kidney transplantation, the levels of PRF1 and GZMB are significantly elevated in the urine and peripheral blood [34]. In this study, a significant upregulation of *prf1* and *gzmb* was detected in the transplanted testes of spotted sea bass. This confirms that a strong immune rejection response occurs in the spotted sea bass testes during the early stage of transplantation.

Studies have indicated that GZMB can induce target cell apoptosis through multiple pathways. Initially, GZMB can directly or indirectly trigger apoptosis in target cells by activating the caspase family apoptosis cascade [35]. GZMB is capable of directly cleaving procaspase 3 or procaspase 8 to activate the function of *casp3*. However, in spotted sea bass, GZMB did not successfully activate *casp3* in fat greenling spermatogonia. Instead, an upregulation of *casp7* expression was observed. Additionally, GZMB can induce target cell apoptosis by activating the mitochondrial apoptosis pathway [36]. This is achieved by cleaving the BID protein to activate *bax*/*bak*, promoting an increase in mitochondrial membrane permeability and triggering a series of apoptotic signals. In the transplanted fat greenling spermatogonia, we observed an upregulation of *bax* and *bid* expression and a downregulation of *bcl-2*, indicating that GZMB mainly induces apoptosis in the transplanted cells through the mitochondrial apoptosis pathway.

In addition, significant expression of immune-tolerance-related genes was also detected in the transcriptome data of transplanted fat greenling spermatogonia. The *lck* gene, known as lymphocyte-specific protein tyrosine kinase, encodes a non-receptor tyrosine kinase of the Src family tyrosine kinases [37]. Research by Stirnweiss demonstrated that activation of T cells induces a conformational change in *lck*, leading to enhanced kinase activity [38]. This regulation of *lck* activity is crucial for recognizing foreign antigens without attacking non-foreign tissues, thereby maintaining immune tolerance. Zeta-chain-associated protein kinase 70 (zap-70), a critical tyrosine kinase in the T cell receptor (TCR) signaling pathway, operates alongside *lck* in the early events of TCR signaling, ultimately activating T cells [39]. Furthermore, *zap-70* is involved in regulating the immune response of T cells by controlling the intensity and timing of signaling, ensuring appropriate immune responses and avoiding immunopathological processes due to overactivation [40]. Our study found significant upregulation of *lck* and *zap-70* in transplanted fat greenling spermatogonia, indicating the pronounced impact of the immune attack from spotted sea bass and the initiation of an immune response. Moreover, the *hla-drb1* gene, which is vital within the human major histocompatibility complex (MHC) class II region and plays a significant role in antigen presentation, was notably upregulated in the transplanted cells [41]. This observation indicates the potential initiation of a receptor-based immune response in the donor.

The effective establishment of donor germ cell gametogenesis within the recipient testis relies on the integrity of the donor germ cell niche. Previous studies have widely acknowledged the existence of the blood–testis barrier (BTB) within the testis, positing that germ cells entering the BTB are shielded from immunological attacks [42,43]. This study opted to use busulfan for preparing spotted sea bass transplant recipients. Busulfan is an alkylating agent capable of inducing cross-linking reactions in cellular DNA, thereby hindering DNA replication processes and inhibiting cell proliferation [44,45]. It is widely used in the field of cancer treatment [46,47]. However, busulfan exhibits significant toxicity towards male germ cells, effectively destroying reproductive cells within the male testes and potentially leading to infertility [48]. Leveraging this characteristic, busulfan can eradicate endogenous reproductive cells in males, showing efficacy in preparing recipients for spermatogonia transplantation. The use of busulfan-prepared transplantation recipients has been successfully established in various fish species, with the production of donor-derived sperm observed weeks to months after transplantation [5,6,8]. In addition to busulfan treatment, triploid breeding [49] and the knockout or knockdown of the *dnd* gene [7] are commonly used for preparing sterile fish recipients. However, during the preparation of sterile spotted sea bass recipients, it was decided that the time required for triploid breeding and the knockout or knockdown method is too long, as it takes about 2 years for the spotted sea bass to reach sexual maturity. Therefore, we treated the spotted sea bass testes with busulfan consecutively and observed a significant loss of germ cells and the appearance of large cavities, making them suitable for spermatogonia transplantation. Additionally, research indicates that busulfan can damage the BTB, thereby affecting spermatogenesis [50,51]. Consequently, the failure of colonization might be attributed to busulfan-induced BTB damage, which allows immune cells to infiltrate the seminiferous tubules, leading to acute immunological rejection responses. Future studies could further optimize transplantation conditions and explore immunosuppressive strategies. For instance, administration of immunosuppressive agents such as cyclosporine, azathioprine, and prednisone during transplantation could be investigated to suppress the expression of *prf1* and *gzmb*, thereby enhancing the success rate of heterotransplantation [52,53].

## 4. Materials and Methods

### 4.1. Experimental Animal Materials

The spotted sea bass and fat greenling used in this study were purchased from Yushun Ocean Technology Co., Ltd., in Qingdao, Shandong Province, China. The spotted sea bass were all two years old, selected based on similar body weight (1.5 kg ± 200 g), healthy condition, and absence of visible injuries, totaling 120 male fish. They were fed every two days and reared for a total of seven months. The fat greenling were 1.5 years old. For sampling, fish were first anesthetized by immersion in seawater containing 100 ng/mL MS-222 (Sigma-Aldrich, Darmstadt, Germany). Once fully anesthetized, the ventral cavity was opened by cutting along the genital pore to extract the testes.

### 4.2. Preparation of Sterile Recipients

The preparation of sterile recipients in spotted sea bass was performed using busulfan (Sigma, Germany). Busulfan was dissolved in DMSO to prepare a stock solution of 60 mg/mL. A total of 120 spotted sea bass were divided into two groups: the first group, serving as the control, consisted of 30 fish; the second group, comprising 90 fish, was treated with busulfan. Using a size 6 gavage needle, busulfan was injected into the gonads of the adult spotted sea bass according to the specific weight of each individual. The injection was performed slowly through the reproductive pore, with a total dosage of 30 mg/kg. Injections were administered every two weeks, for a total of five injections. Testis samples were collected after the last injection for section and RNA extraction, to quantitatively assess the effectiveness of the busulfan treatment.

### 4.3. Testis Sectioning and Hematoxylin and Eosin (H and E) Staining

Testicular tissue samples were fixed with 4% paraformaldehyde (PFA, Catalog No. P1110, Solarbio, Beijing, China) and dehydrated through a graded methanol series. Subsequently, the tissues were cleared in anhydrous ethanol and xylene before being embedded in paraffin wax. The paraffin blocks were removed from the embedding cassettes and sectioned manually using a microtome, with tissue thickness set at 4 μm. The sections were floated in 50 °C ddH_2_O to fully expand, then carefully lifted onto slides, which were dried overnight at 37 °C. The slides were sequentially treated with xylene (twice), anhydrous ethanol (twice), each for 5 min, followed by rehydration through a graded ethanol series of 95%, 70%, 50%, and 30%, each for 2 min, and finally rinsed in ddH_2_O. Hematoxylin staining solution (Catalog No. G1140, Solarbio, Beijing, China) was applied evenly to the tissue sections for approximately 10 s before excess stain was discarded, and the slides were rinsed in flowing ultrapure water to remove floating color. The tissues were then treated with a graded ethanol series of 30%, 50%, 70%, and 95% for two minutes each, stained in eosin staining solution (Catalog No. G1100, Solarbio, Beijing, China) for approximately 30 s, and briefly immersed in 95% ethanol to wash away floating color. This was followed by sequential treatments in anhydrous ethanol (twice) and xylene (twice), each for 5 min, before mounting with neutral balsam. After the balsam had dried, the slides were examined and photographed under a microscope.

### 4.4. Testicular Tissue RNA Extraction, cDNA Template Synthesis, and qRT-PCR

According to the manufacturer’s instructions, RNA from testicular tissues was extracted using Direct-zol RNA Miniprep Kits (Catalog No. tr205, genstone biotech, Beijing, China). Following the manufacturer’s instructions, cDNA synthesis of the extracted RNA was conducted using the All-In-One 5X RT MasterMix (Catalog No. G490, Applied Biological Materials, Richmond, British Columbia, Canada). Quantitative real-time PCR (qRT-PCR) analyses were performed using 2X Universal SYBR Green Fast qPCR Mix (Catalog No. RK21203, ABclonal, Wuhan, China). The specific usage was taken from the manufacturer’s instructions. The primer design was conducted using the Integrated DNA Technologies (IDT) website (https://sg.idtdna.com/pages (accessed on 4 May 2023)), primarily targeting the 3′ region of mRNA sequences with a total length ranging from 150 to 250 bp. Specifically, *rpl17* was chosen as the reference gene for the fat greenling, while *β-actin* served as the reference gene for the spotted sea bass. The primers used are listed in Table S1. The specific reaction conditions were as follows: pre-denaturation at 95 °C for 5 min; denaturation at 95 °C for 15 s; annealing at 60 °C for 45 s, for a total of 40 cycles. Data analysis and graphical representation were performed using SPSS (Version 20, IBM, Armonk, NY, USA), Microsoft Excel (Version 16.0, Microsoft Corporation, Redmond, WA, USA), and GraphPad Prism 7 (GraphPad Software, San Diego, CA, USA). Independent sample *t*-tests were employed for comparisons between two groups of data. One-way analysis of variance (ANOVA) was utilized for comparisons involving three or more groups of data. Significance was considered when *p* < 0.05, indicating a significant difference, and when *p* < 0.01, indicating a highly significant difference between the data sets.

### 4.5. Isolation, Purification, and PKH26 Staining of Fat Greenling Spermatogonia

Spermatogonia were obtained from the testes of fat greenling through enzymatic digestion [5]. Briefly, testes were dissociated in Leibovitz L-15 medium (L-15, VivaCell, Shanghai, China) containing 0.25% trypsin (Gibco, Grand Island, NY, USA) at 25 °C for 30 min. Subsequently, the dispersed testicular tissue was incubated with 10 mg/mL collagenase IV under the same conditions for 1 h. Collagenase activity was neutralized using an equal volume of fetal bovine serum (FBS, Gibco, Grand Island, NY, USA). The cell suspension was filtered through a 40 µm cell strainer and centrifuged at 200× *g* for 5 min (twice), then resuspended in L-15 medium. Enriched spermatogonia suspensions were obtained by Percoll (Sigma-Aldrich, Darmstadt, Germany) gradient centrifugation, following the method described by [54]. Subsequently, spermatogonia were stained with PKH26 (Sigma-Aldrich, Darmstadt, Germany) and injected into the gonads of busulfan-treated spotted sea bass via the genital pore.

### 4.6. Experimental Design for Heterotransplantation

Three groups were designed for the study. Control Group 1: no treatment, totaling 30 individuals (con); Control Group 2: busulfan-treated but only injected with L-15 medium, without spermatogonia transplantation, totaling 30 individuals (un-injected group, ni); Experimental Group: busulfan-treated and transplanted with fat greenling spermatogonia, totaling 60 individuals (spermatogonial stem cell transplant, SSCT). Spermatogonia stained with PKH26 were injected using a size 6 gavage needle for mice, with an average of 750 μL of cells injected per individual, where the cell density was 10^7^ cells/mL. The cells were injected into the spotted sea bass gonads through the reproductive pore. Testis samples from experimental group were collected 1 day (SSCT1d) and 10 days (SSCT10d) post-transplantation for section observation of the donor spermatogonia status in the experimental group. At one day post-transplantation, three testis samples were collected from each of the con, ni, and SSCT1d groups. At 10 days post-transplantation, three SSCT10d samples were collected. These samples were used for RNA extraction and RNA-seq analysis.

### 4.7. Immunofluorescence of Testicular Tissue

Testicular tissue sections were dewaxed and rehydrated, followed by Sodium Citrate Antigen Retrieval Solution (Solarbio, Beijing, China) at 95 °C for 15 min. Tissue permeabilization was conducted using phosphate buffered saline (PBS) containing Triton X-100. Tissue sections were blocked with 10% goat serum diluted in PBS at room temperature for 30 min. The VASA antibody (Gene Tex, Irvine, CA, USA) was diluted 1:500 and incubated at room temperature for 30 min, followed by incubation at 4 °C for 24 h, then returned to room temperature for an additional 30 min. Sections were washed five times with PBS for 3 min each. Goat Anti-Rabbit IgG/FITC (Solarbio, Beijing, China) was diluted 1:200 and incubated at room temperature for 1 h. This was followed by five PBS washes for 3 min each. DAPI staining (Solarbio, Beijing, China) solution was diluted 1:1000 and applied for 1 min, then sections were washed 3–5 times with PBS. Slides were mounted with 50% glycerol and observed and photographed under a fluorescence microscope.

### 4.8. Detection of Fat Greenling Germ Cells in the Testis of Spotted Sea Bass during the Reproductive Period

Intramuscular injections of HCG (500 IU) and LRH-A (250 μg/kg) were administered to induce sperm production in the testis of spotted sea bass during the reproductive period. DNA from the testes post-induction (*n* = 16) was extracted using TIANamp Marine Animals DNA Kit (Catalog No. DP324, TIANGEN, Beijing, China). Additionally, six individuals each of spotted sea bass and fat greenling were selected, and DNA was extracted from their testicular tissues. Primers capable of specifically amplifying target sequences in spotted sea bass and fat greenling, respectively, were screened. The specificity of the known fat greenling microsatellite sequence Heot42 [55] primers was validated through PCR, ensuring amplification occurred only in fat greenling DNA. Furthermore, primers for the *dmrt1* gene, designed based on the spotted sea bass genome sequence, were developed to specifically amplify only in spotted sea bass DNA, as shown in Table S2.

### 4.9. Quality Control and Data Processing

RNA was extracted from testicular tissue samples collected from four treatment groups: control, ni, SSCT1d, and SSCT10d. Three libraries were constructed for each group, resulting in a total of twelve libraries. Each library was derived from the testis of an individual subject. The methods for library construction and sequencing were conducted as follows: a specific quantity of total RNA was extracted, followed by the capture of mRNA from total RNA using oligo dT magnetic beads. The mRNA sequences were fragmented and subjected to random primer and dUTP addition, facilitating the synthesis of double-stranded cDNA. The ends of the cDNA double strands were repaired, ligated, and enzymatically digested, followed by PCR amplification and product recovery. The quality of the library was assessed. Library products underwent circularization treatment, followed by rolling circle replication, resulting in the formation of DNA nanoballs, which were subsequently sequenced on the DNBSEQ platform. The aforementioned procedures were conducted by BGI Genomics.

Following sequencing and quality control, a total of 283,622,077 clean reads were obtained from the extracted RNA. The quality of the raw reads was assessed using FastQC (http://www.bioinformatics.babraham.ac.uk/projects/fastqc/ (accessed on 10 January 2023)). Adapters and low-quality bases were trimmed by Trimmomatic v0.39 (http://www.usadellab.org/cms/?page=trimmomatic (accessed on 10 January 2023)). All filtered clean reads from con, ni, SSCT1d, and SSCT10d groups were mapped to the *L. maculatus* reference genome (NCBI, GCA_004023545.1) using HISAT2 v2.1.0 (https://daehwankimlab.github.io/hisat2/ (accessed on 31 January 2023)) and aligned with the reference transcript sequence by StringTie v2.1.3 (http://ccb.jhu.edu/software/stringtie/ (accessed on 10 February 2023)).

The analysis of the expression pattern of fat greenling spermatogonia in the mixed transcriptome was performed as follows. Initially, the clean reads from the fat greenling testes were assembled using Trinity [56] to obtain a reference sequence for subsequent analyses. Subsequently, the clean reads from ni, SSCT1d, and SSCT10d samples were aligned to the reference sequence using bowtie2, setting the parameter mismatch to 0 (the default parameter for bowtie2), and quantification was performed using the RSEM (https://github.com/deweylab/RSEM (accessed on 10 February 2023)) [57]. The FPKM (expected number of fragments per kilobase of transcript sequence per millions base pairs sequenced) standard was utilized to quantify gene expression levels.

### 4.10. Differentially Expressed Gene Analysis and Functional Enrichment

Reads with TPM (transcripts per million) or FPKM values less than 1 across all twelve samples in the four groups were eliminated. DEseq2 v1.30.0 was used to determine the DEGs (http://www.bioconductor.org/packages/release/bioc/html/DESeq2.html (accessed on 21 February 2023)). To determine the differentially expressed genes (DEGs) across various groups, the criteria were established as follows: genes exhibiting a log_2_FoldChange ≥ 1 or log_2_FoldChange ≤ −1, coupled with a *q*-value < 0.05, were classified as DEGs. DEGs were subjected to enrichment analysis for Gene Ontology (GO) terms and Kyoto Encyclopedia of Genes and Genomes (KEGG) pathways utilizing the DAVID tool. The outcomes were visualized using bubble charts and histograms created in RStudio. To elucidate the overall similarities and disparities in the expression profiles across all samples, principal component analysis (PCA), volcano plots, and heatmaps were employed. These visualizations and analyses were conducted using appropriate R packages.

### 4.11. In Situ Hybridization

The primer sequences for *prf1* and *gzmb* probes are shown in Table S3. Testis sections were dewaxed and rehydrated, followed by two washes in PBS and digestion with 2 µg/mL proteinase K. The enzyme reaction was terminated with triethanolamine solution; sections were then washed twice in PBS and fixed with PFA for 20 min. After another two washes in PBS, sections were pre-hybridized in hybridization solution at 60 °C for 4 h. Probes were diluted to 1 ng/μL and incubated at 60 °C for 12 h. Probe washing involved a series of washes in a mix of 2×SSC and hybridization solution, 2×SSC, 1×SSC, and 0.2×SSCT. Sections were then washed twice in maleic acid and blocked in Blocking Buffer (Roche, Basel, Switzerland) for 2 h. Anti-Digoxigenin-AP Fab fragments (Roche, Basel, Switzerland) were diluted 1:2000 and incubated at 4 °C for 16 h. Antibody washes included two maleic acid washes followed by eight PBS washes. For color development, sections were washed twice in AP Buffer (Roche, Basel, Switzerland), then color substrate was diluted 1:50, with the reaction stopped in PBS after 2 h. Tissues were stained with neutral red, dehydrated in a graded ethanol series (70%, 90%, absolute ethanol twice), cleared in xylene (twice), and mounted with neutral balsam. After drying, sections were examined and photographed under a microscope.

### 4.12. Bimolecular Fluorescence Complementation and Immunoprecipitation

Primers were designed based on the open reading frames (ORFs) of the *prf1* and *gzmb* genes for the construction of bimolecular fluorescence complementation (BiFC) vectors pBiFC-*prf1*-VC155 and pBiFC-*gzmb*-VN173, as well as immunoprecipitation (Co-IP) vectors *prf1*-pEGFP-N1, *gzmb*-pEGFP-N1, and *gzmb*-pcDNA3.1-3×FLAG. The sequences of primers, including restriction sites, are presented in Supplementary Table S4. Using testicular cDNA as a template, the full-length sequences of the *prf1* and *gzmb* genes were amplified via PCR, followed by the recovery of the target fragments through agarose gel electrophoresis. Based on the restriction sites of the primers, the target fragments and the empty plasmids pBiFC-VC155, pBiFC-VN173, pEGFP-N1, and pcDNA3.1-3×FLAG were digested with dual enzymes. The digested target fragments and linearized vectors were recovered through agarose gel electrophoresis, after which the target fragments were ligated to the linear vectors. Following transformation, plating, and sequencing, plasmids were extracted.

In the BiFC experiment, HEK-293T cells were cultured in 24-well culture plates. Four different transfection groups were established: transfection with the pBiFC-*prf1*-VC155 vector, transfection with the pBiFC-*gzmb*-VN173 vector, co-transfection with both pBiFC-*prf1*-VC155 and pBiFC-*gzmb*-VN173 vectors, and a positive control group, co-transfection with pBiFC-bFos-VC155 and pBiFC-bJun-VN173 vectors. At 48 h after transfection, cells were stained with DAPI and photographed under a fluorescence microscope.

In the Co-IP experiment, HEK-293T cells were cultured in 60 mm culture dishes. Three distinct transfection groups were established: one transfected with the *prf1*-pEGFP-N1 vector, another with the *gzmb*-pcDNA3.1-3×FLAG vector, and a third group co-transfected with both *prf1*-pEGFP-N1 and *gzmb*-pcDNA3.1-3×FLAG vectors. After 72 h of transfection, the culture medium was discarded, and cells were washed twice with 1 mL of PBS, followed by the use of BeaverBeads™ Protein A/G Immunoprecipitation Kit (Catalog No. 22202-20, Beaver, Suzhou, China) to isolate the target proteins.

### 4.13. Western Blot

Protein gels were prepared using the Omni-Easy™ One-Step PAGE Gel Fast Preparation Kit (Catalog No. PG212, EpiZyme, Shanghai, China), with the loading amount adjusted to 20 μg based on the concentration of the protein samples. The PAGE gel was run at 150 V at 4 °C. The PAGE gel was rinsed with wet transfer buffer (containing 200 mL methanol, 14.4 g glycine, and 3.03 g Tris per liter) and then carefully placed on a sponge pad for western blot. A polyvinylidene difluoride (PVDF) membrane, cut slightly larger than the gel and activated by methanol, was placed over the gel, followed by another layer of sponge pad. The membrane was transferred in wet transfer buffer at 200 mA for 2 h. The membrane was blocked with 5% skim milk (diluted in TBS, pH = 7.6) at room temperature for 2–3 h. EGFP Rabbit pAb and Flag Tag Recombinant Mouse mAb (Bioss, Beijing, China) were diluted 1:4000 and incubated overnight at 4 °C, followed by 1 h of rewarming at room temperature. The PVDF membrane was washed with TBS six times for 5 min each. Goat Anti-Rabbit IgG and Goat Anti-Mouse IgG (CWBIO, Shanghai, China) were diluted 1:5000 and incubated at room temperature for 50 min. The PVDF membrane was washed with TBS six times for 5 min each. According to the manufacturer’s instructions, an Omni-ECL™ Femto Light Chemiluminescence Kit (Catalog No. SQ201, EpiZyme, Shanghai, China) was used for developing the PVDF membrane. After 30 seconds of development, images were captured using a gel imaging system.

### 4.14. The Culture and Transfection of Spermatogonia from the Fat Greenling

The culture and transfection of spermatogonia from the fat greenling were conducted following standardized protocols. The spermatogonia were cultured in L-15 medium supplemented with 10 ng/mL bovine insulin (Solarbio, Beijing, China), 1nM sodium pyruvate (Solarbio, Beijing, China), 1% fish serum, 10 ng/mL glial cell line-derived neurotrophic factor (GDNF, ABclonal, Wuhan, China), 10 ng/mL basic fibroblast growth factor (bFGF, ABclonal, Wuhan, China), and 5% fetal bovine serum (FBS).

For the transfection procedure, cells at the optimal confluence were transfected with *prf1*-pEGFP-N1 and *gzmb*-pEGFP-N1 plasmids in the experimental group, and with the empty pEGFP-N1 plasmid in the control group, using Lipofectamine 3000 Transfection Reagent (Catalog No. L3000015, Invitrogen, Waltham, MA, USA) according to the manufacturer’s instructions. Post-transfection, cells were maintained under standard culture conditions and monitored for expression of the transgene. Total RNA was extracted from spermatogonia of both the experimental and control groups, and cDNA was synthesized. The expression of *prf1*, *gzmb*, and apoptosis-related genes was analyzed by qRT-PCR (following the procedure described in Section 4.4). The primer sequences are listed in Table S1.

## 5. Conclusions

The transplantation of fish spermatogonia is not only significant for understanding the reproductive regulation mechanisms of fish, but also holds immense potential for biodiversity conservation, endangered species reproduction, and the aquaculture industry. In this study, we explored for the first time the factors affecting the transplantation efficiency of spermatogonia between different orders of fish. Our findings demonstrate that a significant immune response occurred in the testes of spotted sea bass after the transplantation of fat greenling spermatogonia, and the transplanted cells underwent extensive apoptosis. Moreover, spotted sea bass PRF1 and GZMB played crucial roles in the acute immune rejection during heterotransplantation. These results highlight the important role of the immune system in the transplantation of fish spermatogonia. In light of our findings, future research should focus on exploring how to suppress the host’s immune response, thereby facilitating the colonization of xenogeneic spermatogonia.

## Data Availability

All data supporting the findings of this study are available within the article and its Supplementary Materials. Additionally, the transcriptomic raw data have been deposited in the NCBI, with the BioProject ID PRJNA1091861.

## Supplementary Materials

The following supporting information can be downloaded at: https://www.mdpi.com/article/10.3390/ijms25105157/s1.
