# Supplementary material for: Immune Rejection Mediated by prf1 and gzmb Affects the Colonization of Fat Greenling (Hexagrammos otakii) Spermatogonia in Heterotransplantation
Source: Int J Mol Sci. 2024 May 9;25(10):5157. doi: 10.3390/ijms25105157 (PMC11121654; doi:10.3390/ijms25105157)
Supplement: Supplementary file 1 [file ijms-25-05157-s001.zip › Tables S1-S4.pdf]

Primer name	Sequence（5’ to 3’）	Function
Lma-vasa-qPCR-fw	CCCAACATTGACCTGAGTTA	qRT-PCR
Lma-vasa-qPCR-rv	GTCCGGCCTGAAACAAA	
Lma-sycp3-qPCR-fw	GTTAACGCAGCGACTTCT	qRT-PCR
Lma-sycp3-qPCR-rv	CTCTCCCACTGACATGAAAC	
Lma-prf1-qPCR-fw	GCATCCCTCAGCATCAAA	qRT-PCR
Lma-prf1-qPCR-rv	AAGCGAGTCCAGGGAATA	
Lma-hla-dpb1-qPCR-fw	TTTCCGCTTCGCCATTT	qRT-PCR
Lma-hla-dpb1-qPCR-rv	AGCGTACTCGGGACTTT	
Lma-gzmb-qPCR-fw	TGTCACCATGTTTGTCCAG	qRT-PCR
Lma-gzmb-qPCR-rv	GAAGCCGCCACAGTATTT	
Lma-cd8a-qPCR-fw	CAACCGCAACCCATTACT	qRT-PCR
Lma-cd8a-qPCR-rv	CGACCAGTCGAGTTACTTTC	
Lma-cd3e-qPCR-fw	AACAGCAGGACAGGATAGA	qRT-PCR
Lma-cd3e-qPCR-rv	ACTGTGAGCACCACAAAC	
Lma-cd56-qPCR-fw	TCCTTGTGACGGGTGATA	qRT-PCR
Lma-cd56-qPCR-rv	CTGAGGCAGTCTGGTTATTC	
Lma-il8l-qPCR-fw	AGACTTCTCGGTCCACAA	qRT-PCR
Lma-il8l-qPCR-rv	AAGGCACAGCTTCACATC	
Lma-eif4-qPCR-fw	CCGGAGGTACGAGAATCA	qRT-PCR
Lma-eif4-qPCR-rv	GCTGTGGTCGGTGTTATT	
Lma-lsm3-qPCR-fw	CGTGAAGATGAGGAACGAC	qRT-PCR
Lma-lsm3-qPCR-rv	TCCTCTTGGTGGACTTGTA	
Lma-dmrtb1-qPCR-fw	TGCACCTCTGCCTCTTAT	qRT-PCR
Lma-dmrtb1-qPCR-rv	GGTTGAAGGGTGAAGAAGAC	
Lma-rsrp1-qPCR-fw	CCCACTCCAGGTCTTCTAA	qRT-PCR
Lma-rsrp1-qPCR-rv	GCTTCACGCTCTCACAAA	
Lma-cox3-qPCR-fw	AGGGCTTCGATACGGTATAA	qRT-PCR
Lma-cox3-qPCR-rv	ACACCGGAGGCTAGTAAA	
Lma-hamp-qPCR-fw	CTCGTGCTCGCCTTTATT	qRT-PCR
Lma-hamp-qPCR-rv	TTCTGCCTGATGTGATTGG	
Lma-rbp4-qPCR-fw	TCCTCAACAACTGGGAAATG	qRT-PCR
Lma-rbp4-qPCR-rv	AGTGGACGGCGTAGTTAT	
Hot-lck-qPCR-fw	TTACCCAGGAGCCAATCT	qRT-PCR
Hot-lck-qPCR-rv	TCAGGTCTCGGTGAATGT	
Hot-zap70-qPCR-fw	ACAGGAGTCGGGTAGTTT	qRT-PCR
Hot-zap70-qPCR-rv	GGAATGCTGGGTGTCTTT	
Hot-hla-drb1-qPCR-fw	ATGAAGGCTCAGAAGGAGA	qRT-PCR
Hot-hla-drb1-qPCR-rv	TGGCAGCTCATCAGTAGA	
Hot-casp3-qPCR-fw	ATGAGGGCGTGTTCTTTG	qRT-PCR
Hot-casp3-qPCR-rv	TCCTGGTAGTGCCATCTT	
Hot-casp7-qPCR-fw	TCTACGGTGCCAGGTTAT	qRT-PCR
Hot-casp7-qPCR-rv	CTGGCGGTCAGTTGAAAT	
Hot-bax-qPCR-fw	AGATCGCAGACGAGATGA	qRT-PCR
Hot-bax-qPCR-rv	TGTAGGCCAGATGGAAGA	
Hot-bcl-2-qPCR-fw	ACAACGCACAGGCTTAAT	qRT-PCR
Hot-bcl-2-qPCR-rv	CCTAAAGGATCCGGGAAATG	
Hot-bnip2-qPCR-fw	CTATGGAGACGGAACGAATG	qRT-PCR
Hot-bnip2-qPCR-rv	GTGGTTGATGGTGGTGTAA	
Hot-bid-qPCR-fw	GCTCCACCCACACATATTC	qRT-PCR
Hot-bid-qPCR-rv	ACCTACCTACCTACCTACCT	
Lma-βactin-qPCR-fw	CAACTGGGATGACATGGAGAAG	qRT-PCR
Lma-βactin-qPCR-rv	TTGGCTTTGGGGTTCAGG	
Hot-rpl17-qPCR-fw	ACCGAGAAGGAGCAGAT	qRT-PCR
Hot-rpl17-qPCR-rv	GGTTCAGAACTTGGTGTTTAT	

Table S1. Primers used for qRT-PCR analysis.

Primer name	Sequence（5’ to 3’）
Hot-Heot42-fw	CAGAAAACGTTGCCATGAAA
Hot-Heot42-rv	GTGAAACGAGCCCCCTACAG
Lma-dmrt1-fw	CCAACGTCTGTCCACATTTA
Lma-dmrt1-rv	CCAGCACACATTCACATTTAC

Table S2. Primers used for Heot42 and *dmrt1*.

Primer name	Sequence（5’ to 3’）	Function
Lma-prf1-ISH-T7	TAATACGACTCACTATAGGGAGACATCTTGTCTGCCACTAC	ISH
Lma-prf1-ISH-SP6	ATTTAGGTGACACTATAGAAGAGGGCTTTACTGAGGCTTAC	
Lma-gzmb-ISH-T7	TAATACGACTCACTATAGGGAGATAAGAGCAGAGGACATCAA	ISH
Lma-gzmb-ISH-SP6	ATTTAGGTGACACTATAGAAGAGACATGGAAAGCGCTAATAC	

Table S3. Primers used for *in situ* hybridization.

Primer name	Sequence（5’ to 3’）	restriction sites
Lma-prf1-VC155-fw	AGATCTCTATGTTTCTGTTGAATATTTGC	Bgl II
Lma-prf1-VC155-rv	GGTACCTATCACATCAAACTCTTGACTC	Kpn I
Lma-gzmb-VN173-fw	AAGCTTATGTTTGTCCAGTGTAAA	Hind III
Lma-gzmb-VN173-fw	GGTACCGAGCGCTTTCCATGTTTCTT	Kpn I
Lma-prf1-N1-fw	AGATCTCATGTTTCTGTTGAATATTTGC	Bgl II
Lma-prf1-N1-rv	GGTACCGTTATCACATCAAACTCTTGAC	Kpn I
Lma-gzmb-N1-fw	AGATCTCATGTTTGTCCAGTGTAAATTG	Bgl II
Lma-gzmb-N1-rv	GGTACCGTGCGCTTTCCATGTTTCTTC	Kpn I
Lma-gzmb-3xFLAG-fw	CTCGAGATGTTTGTCCAGTGTAAATTGG	Xho I
Lma-gzmb-3xFLAG-rv	GGTACCGAGCGCTTTCCATGTTTCTTC	Kpn I

Table S4. Primers used for BiFC and Co-IP.
