# Supplementary figures and images for: Immune Rejection Mediated by prf1 and gzmb Affects the Colonization of Fat Greenling (Hexagrammos otakii) Spermatogonia in Heterotransplantation
Source: Int J Mol Sci. 2024 May 9;25(10):5157. doi: 10.3390/ijms25105157 (PMC11121654; doi:10.3390/ijms25105157)

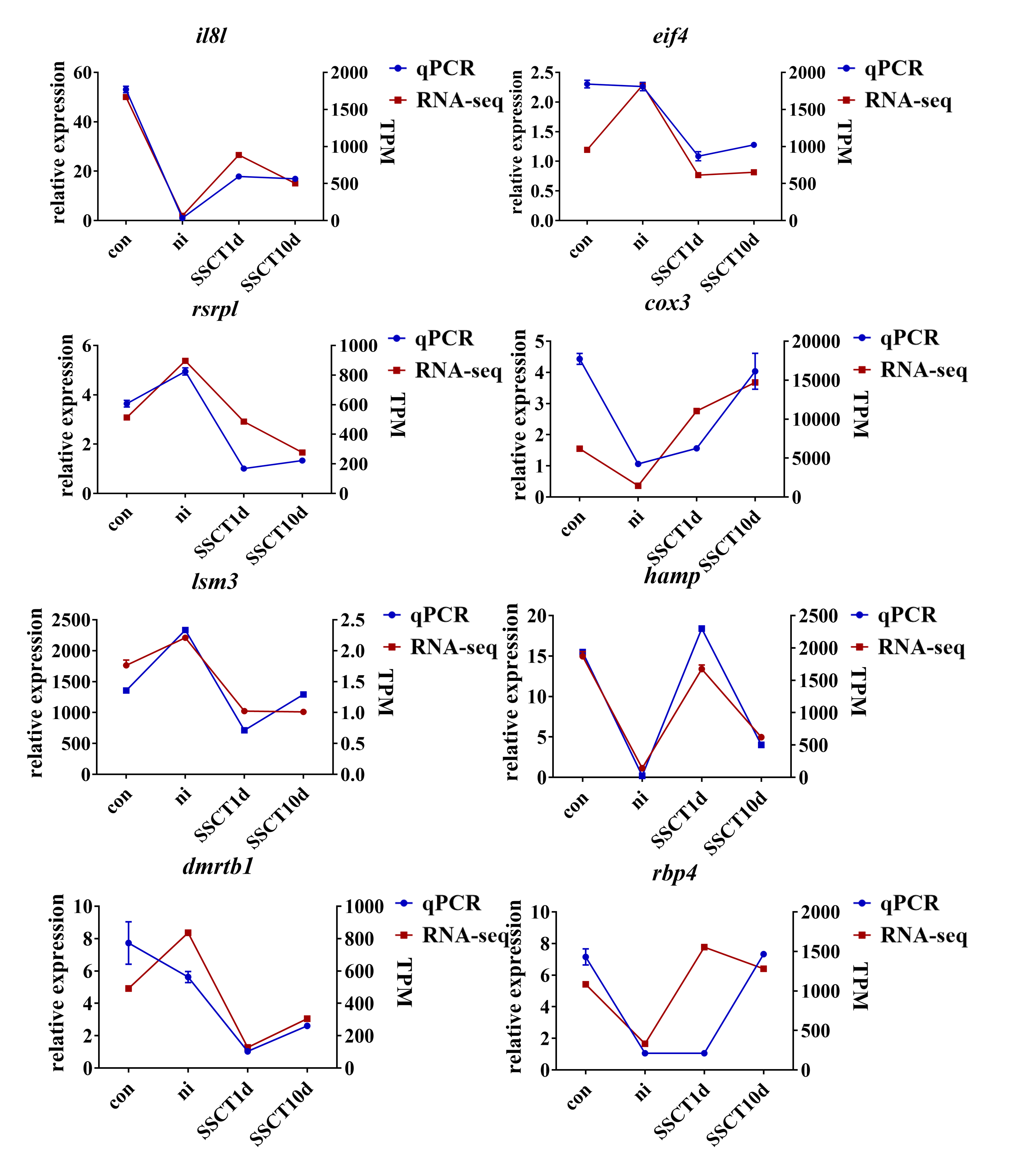

Supplement: Supplementary file 1 [file ijms-25-05157-s001.zip › Figure S1. Verification of differential genes expression before and after busulfan treatment of the spotted sea bass testis.tif]

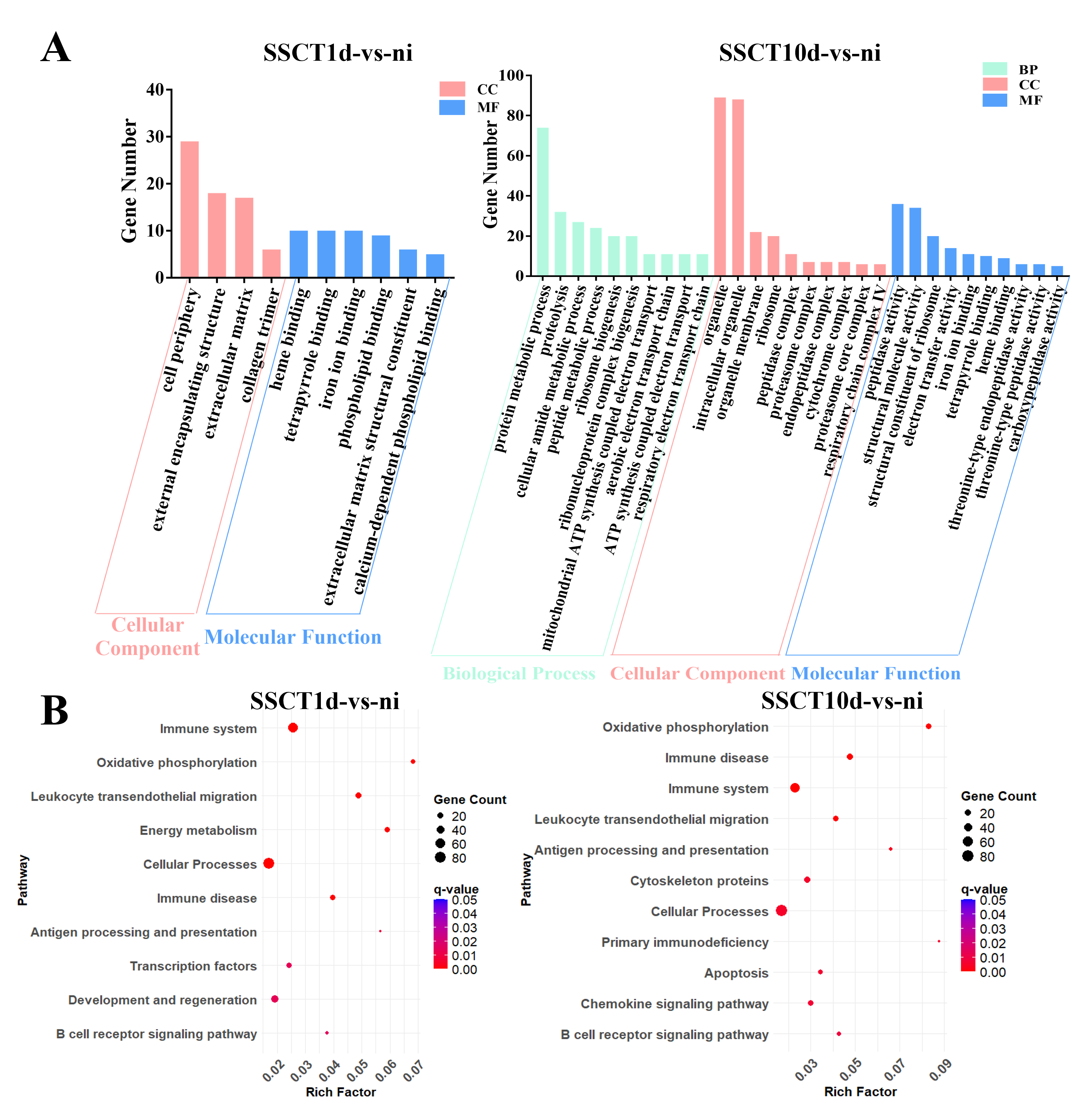

Supplement: Supplementary file 1 [file ijms-25-05157-s001.zip › Figure S2. GO and KEGG enrichment analysis of differentially expressed genes in fat greenling spermatogonia after hetertransplantation into spotted sea bass testes.tif]

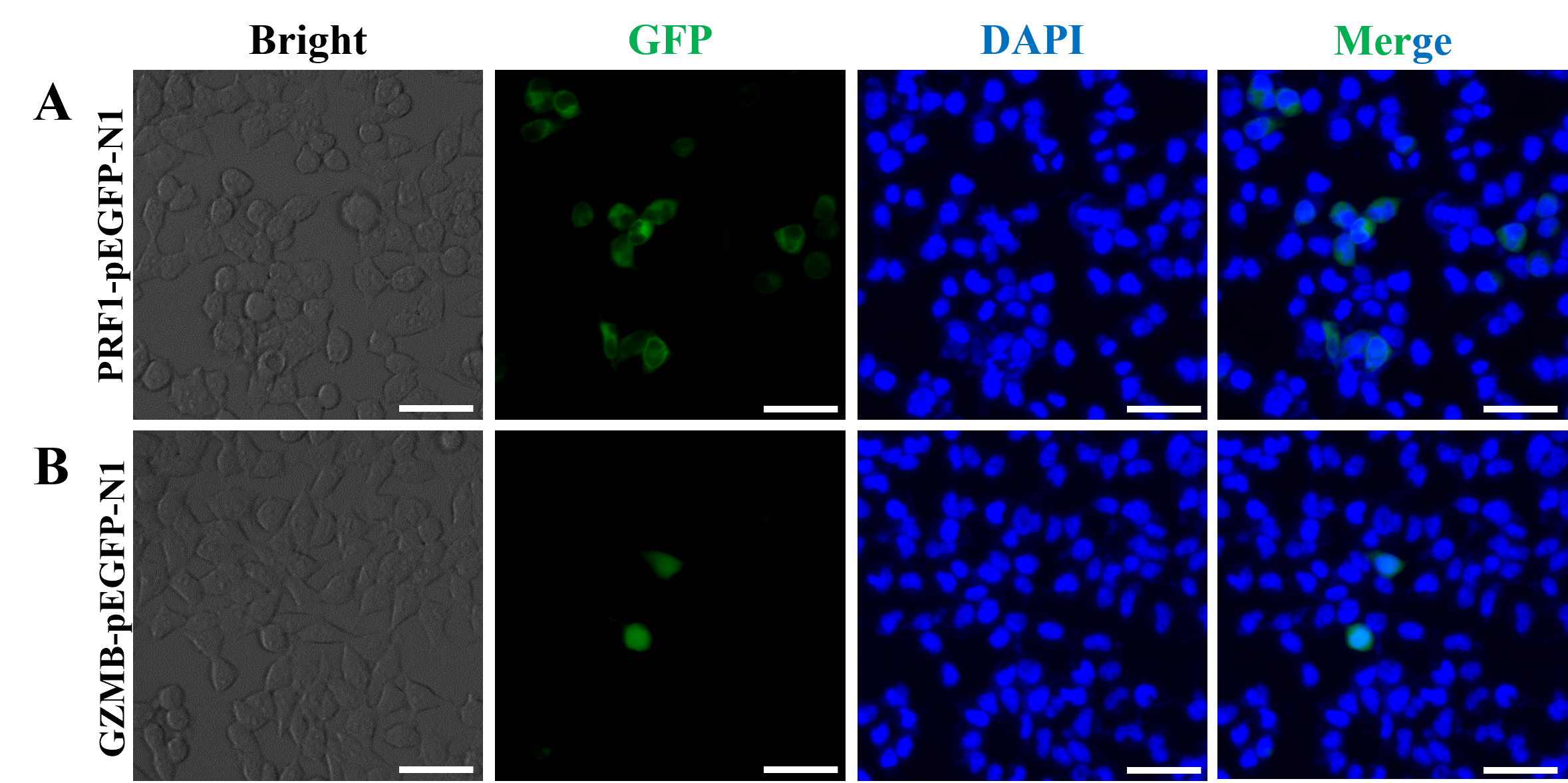

Supplement: Supplementary file 1 [file ijms-25-05157-s001.zip › Figure S3. Subcellular localization of PRF1 and GZMB from spotted sea bass in HEK-293T cells.tif]
